# SLC31A1 knockdown mitigates post-MI heart failure via regulation of copper metabolism

**DOI:** 10.3389/fimmu.2026.1707203

**Published:** 2026-02-23

**Authors:** Qi Wei, Huanyu Zhou, Jie Sun, Ling Qin

**Affiliations:** 1 Department of Cardiology, First Hospital of Jilin University, Changchun, Jilin, China; 2 Department of Integrated Cardio-Oncology, Jilin Province Cancer Hospital, Changchun, Jilin, China; 3 Department of Geriatrics, First Hospital of Jilin University, Changchun, Jilin, China

**Keywords:** acute myocardial infarction, apoptosis, copper metabolism, cuproptosis, heart failure, HMGB1, macrophages, NLRP3

## Abstract

**Introduction:**

Cuproptosis due to copper overload is a contributor to the progression of cardiovascular diseases, especially heart failure (HF) after acute myocardial infarction (AMI). Solute carrier family 31 member 1 (SLC31A1) is a major Cu2+ transporter responsible for intracellular Cu2+ uptake. In this study, we investigated the role and detailed mechanism of SLC31A1 in post-AMI HF.

**Methods:**

Mouse left anterior descending coronary artery was ligated to produce an *in vivo* post-AMI HF model. These mice were subjected to treatments with short hairpin RNA targeting SLC31A1, the copper chelator ATTM and the NLRP3 agonist nigericin to elucidate the mechanism of SLC31A1 in post-AMI HF. Additionally, an *in vitro* model of hypoxia was induced in macrophages RAW264.7, which were then treated with small interfering RNA targeting SLC31A1, ATTM and nigericin, and subsequently co-cultured with cardiomyocytes to validate the SLC31A1 mechanism *in vitro*.

**Results:**

SLC31A1 was up-regulated in macrophages of mice with post-AMI HF , while its knockdown prevented cardiomyocyte apoptosis and post-AMI HF. Mechanistically, SLC31A1 knockdown regulated copper metabolism imbalance to reduce macrophage cuproptosis and HMGB1 release, attenuating inflammatory responses and the resultant cardiomyocyte apoptosis. This could be explained by NLRP3 inflammasome inactivation. Meanwhile, ATTM reduced macrophage cuproptosis and cardiomyocyte apoptosis. These results were reproduced in *in vitro* studies. Strikingly, NLRP3/HMGB1 activation in vivo partly abolished SLC31A1 knockdown-induced alleviation of macrophage cuproptosis and cardiomyocyte apoptosis.

**Discussion:**

SLC31A1 plays a disease-promoting role in HF after AMI by activating NLRP3/HMGB1-dependent macrophage cuproptosis, which is expected to be a potential biomarker for HF.

## Introduction

Heart failure (HF) is a clinical syndrome in which the heart is unable to pump sufficient blood to the body because of reduced myocardial force development (1). It is a systemic and multifactorial condition that causes significant morbidity and mortality worldwide, inducing substantial health-care burden (2, 3). Acute myocardial infarction (AMI) stands as a primary cause of HF (4, 5). Copper is an essential micronutrient for a broad range of physiological processes in virtually all cell types, yet it can be toxic at higher concentrations, inducing cell death (6, 7). Copper-induced cell death, named cuproptosis, has become increasingly recognized as a crucial contributor to the pathogenesis and progression of cardiovascular diseases, especially HF (8). This is due to the fact that mitochondria are central mediators of cardiomyocyte survival and death, and meanwhile, cuproptosis exhibits close association with mitochondrial respiration (9). An in-depth understanding of the regulatory mechanisms of cuproptosis in post-AMI HF may be thus useful to guide patient management.

Solute carrier family 31 member 1 (SLC31A1) is an important copper transporter responsible for intracellular copper uptake; evidence has strongly associated SLC31A1 expression with overall survival in several diseases, suggesting its potential utility as a diagnostic biomarker (10, 11). Particularly, SLC31A1 shows an excellent diagnostic property in HF due to its close correlation to energy metabolism and immune activity (12). In addition, SLC31A1 has been found to be predominantly localized in macrophages of atherosclerotic lesions (13). Oxygen limitation, as previously reported, is a trigger for profound alterations in copper homeostasis in RAW264.7 macrophages; further, hypoxia stimulates copper uptake and increases expression of SLC31A1 (14). To this end, it is tempting to speculate that SLC31A1 may participate in HF after AMI by mediating cuproptosis in macrophages. In addition to SLC31A1, NLR pyrin domain containing 3 (NLRP3) may also serve as a predictor of cuproptosis (15). As an innate immune sensor, NLRP3 detects exogenous pathogenic patterns and endogenous damage signals, and then responds by forming the NLRP3 inflammasome, a supramolecular complex that plays a key role in immune monitoring (16). Recent work has illustrated that sustained copper accumulation in BV2 cells activates macrophages to secrete inflammatory cytokines, which is related to activation of the NLRP3 inflammasome (17). Remarkably, the inflammation of the NLRP3 inflammasome is thought to play an important role in the development of HF (18). In contrast, the NLRP3 inflammasome inactivation can delay the release of high-mobility group box 1 protein (HMGB1), interleukin (IL)-1β and IL-18 by macrophages (19). HMGB1, also referred to as amphoterin, is a highly conserved, ubiquitous protein present in almost all eukaryotic cells (20). HMGB1 can be released by cuproptotic cells to initiate inflammation; from a mechanistic perspective, copper accumulation leads to adenosine triphosphate (ATP) depletion and AMP-activated protein kinase (AMPK) activation to provoke HMGB1 phosphorylation, contributing to increased extracellular release of HMGB1 (21). This release induces a robust secretion of an inflammatory cytokine IL-17A, which is an essential mechanism resulting in cardiomyocyte apoptosis and ventricular remodeling following MI (22). In light of these mentioned reports, we hypothesized that SLC31A1 might mediate copper metabolism imbalance and lead to cuproptosis in macrophages by activating the NLRP3 inflammasome, which allowed HMGB1 release, and consequent inflammatory responses, thereby accelerating post-AMI HF. To examine this possibility, we conducted *in vitro* functional and *in vivo* animal experiments.

## Materials and methods

### Ethics statement

The animal study was approved by the Animal Ethics Committee of Jilin Province Cancer Hospital. The study was conducted in accordance with the local legislation and institutional requirements.

### Establishment of the post-AMI HF mouse model

Ninety-six C57BL/6J mice (6–8 weeks old, weighing 22 ± 2 g, half male and half female, Changchun Biological Products Research Institute Co., Ltd., Changchun, Jilin, China) were kept in a standard animal house at 22 ± 2°C under a 12-h light/dark cycle (lights on from 8:00 to 20:00), with ad libitum access to food and water.

The AMI mouse model was generated as previously described (23). In short, mice underwent anesthesia (intraperitoneal injection with 50 mg/kg sodium pentobarbital), and the chest cavity was opened to expose the heart. The pericardium was then opened, and the left anterior descending (LAD) coronary artery was ligated with a 7–0 suture. Three days before the operation, mice were subjected to tail vein injection with 100 μL adenovirus-associated vector expressing a short hairpin RNA (shRNA) against SLC31A1 (sh-SLC31A1; 1 × 10^12^ vg/mL) and its negative control (sh-NC; Hanbio Biotechnology Co., Ltd., Shanghai, China) (24). In addition, 2 h before the operation, mice were intraperitoneally injected with 1 mg/kg of the NLRP3 agonist nigericin (25) (HY-127019, MedChem Express, Monmouth Junction, NJ, USA) and an equal amount of the solvent (Vehicle) (10% dimethyl sulphoxide [DMSO] + 40% PEG300 + 5% Tween-80 + 45% Saline; MedChem Express, Shanghai, China). At 3 days after operation, intragastric administration with 10 mg/kg ammonium tetrathiomolybdate (ATTM, a copper chelator; HY-W076067, MedChem Express, USA) and an equal amount of normal saline (NS) was performed once a day for a total of 3 days (26). For sham operation (Sham), only the chest was opened, but no ligation of the LAD coronary artery was performed. After operation, mice were subcutaneously injected with 0.02 mL of buprenorphine (0.3 mg/mL) for 2 days to relieve pain.

All mice were randomly grouped into the Sham, AMI, AMI + sh-NC, AMI + sh-SLC31A1, AMI + NS, AMI + ATTM, AMI + sh-SLC31A1 + Vehicle and AMI + sh-SLC31A1 + Nigericin groups (n = 12). Afterwards, the corresponding indicators of echocardiography were detected using instruments. Then, mice were anesthetized by intraperitoneal injection with sodium pentobarbital (50 mg/kg). Blood was drawn from the mouse abdominal aorta (approximately 0.8 mL per mouse), half of which was used to extract macrophages and the remaining used to detect serum indicators. Excessive sodium pentobarbital (100 mg/kg) was injected intraperitoneally to euthanize the mice. Next, heart tissues from 6 randomly selected mice of each group were used for 2,3,5-triphenyl tetrazolium chloride (TTC) staining, and those from the other 6 mice for terminal deoxynucleotidyl transferase-mediated dUTP-biotin nick end labeling (TUNEL) staining.

### Echocardiography

As previously described (27), S90 Exp (Sonoscape, Shenzhen, Guangdong, China) was applied for M-mode echocardiography recording, with internal dimensions of the left ventricle during systole and diastole measured, and the left ventricular ejection fraction (LVEF) calculated. Under isoflurane anesthesia, a Millar catheter was inserted into the left ventricle through the right carotid artery of the mice. This catheter was connected to the Data Acquisition System and the data were recorded using AcqKnowledge 3.9 software (BIOPAC Systems Inc., Goleta, CA, USA). When the mice responded to a toe pinch, left ventricle end-diastolic pressure (LVEDP) was recorded and measured.

### TTC staining

Fresh heart tissues were frozen and horizontally cut into five equal sections. Each section was incubated with preheated TTC solution (C0651, Beyotime, Shanghai, China) at 37°C for 10 min. The shape of the heart section was adjusted under a microscope, and maintained by soaking the section in a formalin solution. Images were taken within 24 h and the staining results were quantitatively analyzed using Image Pro Plus 6.0 software (Media Cybernetics, Silver Springs, MD, USA).

### TUNEL staining

Heart tissues were made into paraffin sections, and the subsequent operations were carried out according to the instructions of the TUNEL Assay Kit (C1086, Beyotime). Finally, sections were visualized under a fluorescence microscope (Axioscope 5, Carl Zeiss, Thornwood, NY, USA), and the staining results were analyzed using Image J software (National Institutes of Health, Bethesda, MD, USA).

### Sorting of macrophages

Blood was mixed with phosphate-buffered saline (PBS) at a ratio of 1:1, and slowly added to a clean centrifuge tube containing Ficoll (F2637, Sigma-Aldrich, Steinheim, Germany) along the tube wall so that it floated above the Ficoll layer. Centrifugation was then performed at 400 × g and normal temperature for 40 min. The peripheral blood mononuclear cell (PBMC) layer was collected into a new tube and cells were washed 1–2 times with PBS. After treatment with the red blood cell lysis buffer (C3702, Beyotime), cells were subjected to centrifugation. The obtained pellets were PBMCs, which were then prepared into single-cell suspension in PBS. The suspension was probed with antibodies against allophycocyanin-conjugated F4/80 (1:100, E-AB-F0995E, Elabscience Biotechnology Co., Ltd., Wuhan, Hubei, China) and fluorescein isothiocyanate (FITC)-conjugated CD11b (1:100, E-AB-F1081C, Elabscience) in the dark at 4°C for 30–40 min. Following two washes with the fluorescence-activated cell sorting (FACS) buffer (1 mL each), the suspension was centrifuged at 400 × g for 5 min. The resulting pellets were resuspended in 350 μL FACS buffer, and stained with 4’,6-diamidino-2-phenylindole (C1002, Beyotime), followed by sorting using an FACS instrument (BD Biosciences, Franklin Lakes, NJ, USA) to obtain macrophages.

### Cell treatment and grouping

Mouse macrophages RAW264.7 (CL-0190, Pricella, Wuhan, Hubei, China) were maintained in complete culture medium (CM-0190, Pricella). Mouse cardiomyocytes (CP-M073, Pricella) were placed in complete culture medium (CM-M073, Pricella). All cells were cultured at 37°C with 5% CO_2_. Cells were exposed to hypoxia (1% O_2_) for 24 h in an InVivo2400 hypoxic workstation (Ruskinn Technologies, Bridgend, UK) to establish an *in vitro* hypoxic cell model (the Hypoxia group) (28). The cell model was then treated with 10 μM of the copper chelator ATTM (29),10 μM of the NLRP3 agonist nigericin (30), and 5 μM of the NLRP3 inhibitor MCC950 (31), or an equal amount of DMSO to nigericin for 24 h. Cells in the blank control group (the Blank group) were cultured under normoxic conditions (21% O_2_).

Under instructions of the Lipofectamine 2000 reagent (Invitrogen, Carlsbad, CA, USA), RAW264.7 cells were transfected with plasmids for silencing SLC31A1 (small interfering RNA [si]-SLC31A1) and its NC plasmids (si-NC) (all bought from GenePharma Co., Ltd., Shanghai, China) at a final concentration of 100 nM. Subsequent experiments were carried out 24 h after transfection.

RAW264.7 cells were grouped as follows: the Blank group; the Hypoxia group; the Hypoxia + si-NC group (cells were transfected with si-NC and exposed to hypoxia); the Hypoxia + si-SLC31A1 group (cells were transfected with si-SLC31A1 and exposed to hypoxia); the Hypoxia + si-SLC31A1 + DMSO group (cells were transfected with si-SLC31A1, exposed to hypoxia and treated with an equal amount of DMSO to ATTM); the Hypoxia + si-SLC31A1 + ATTM group (cells were transfected with si-SLC31A1, exposed to hypoxia and treated with ATTM); the Hypoxia + si-SLC31A1 + DMSO group (cells were transfected with si-SLC31A1, exposed to hypoxia and treated with an equal amount of DMSO to nigericin); the Hypoxia + si-SLC31A1 + Nigericin group (cells were transfected with si-SLC31A1, exposed to hypoxia and treated with nigericin); the Hypoxia + MCC950 group (cells were cultured under hypoxic conditions and treated with MCC950); the Hypoxia + si-SLC31A1 + MCC950 group (cells were transfected with si-SLC31A1, exposed to hypoxia and treated with MCC950).

Macrophages (apical chamber) and cardiomyocytes (basolaternal chamber) were co-cultured for 24 h using 0.4 mm Transwell chambers (FTW001, Beyotime). Cardiomyocytes were then harvested and assigned into Co(Blank), Co(Hypoxia), Co(Hypoxia + si-NC), Co(Hypoxia + si-SLC31A1), Co(Hypoxia + si-SLC31A1 + DMSO), Co(Hypoxia + si-SLC31A1 + Nigericin, Co(Hypoxia + MCC950), and Co(Hypoxia + si-SLC31A1 + MCC950) groups.

### Cell counting kit-8 assay

Cell viability was evaluated using the CCK-8 kit (C0037, Beyotime). The specific experimental steps were carried out according to the kit instructions.

### Reverse transcription quantitative polymerase chain reaction

Total RNA was extracted using a Total RNA Isolation Kit (Invitrogen). The extracted RNA was then reverse transcribed into complementary DNA using NovoScript Plus All-in-one 1st Strand cDNA Synthesis SuperMix (abs60076, Absin Bioscience, Shanghai, China). RT-qPCR was conducted using NovoStart SYBR High-Sensitivity qPCR SuperMix (abs60086, Absin Bioscience). The conditions were 30 s of pre-denaturation at 95°C, 40 reaction cycles of 10 s of denaturation at 95°C, 30 s of annealing at 60°C and 3 min of extension at 72°C. β-actin was employed as an internal reference and fold changes were calculated by means of relative quantification (2^-ΔΔCt^ method). The used primer sequences are shown in **Table 1**.

**Table 1:** Primer sequences for RT-qPCR.

Target	Sequence
SLC31A1	Forward: 5’-TATGGGTATGTACCACACGGAC-3’
	Reverse: 5’-GCCATTTCTCCAGGTGTATTGA-3’
NLRP3	Forward: 5’-ATTACCCGCCCGAGAAAGG-3’
	Reverse: 5’-TCGCAGCAAAGATCCACACAG-3’
β-actin	Forward: 5’-GGCTGTATTCCCCTCCATCG-3’
	Reverse: 5’-CCAGTTGGTAACAATGCCATGT-3’

### Western blot

Total protein was extracted using the RIPA lysis buffer (P0013B, Beyotime) containing protease inhibitors (P1005, Beyotime), with the concentration determined by the BCA protein assay kit (P0012, Beyotime). Next, samples were separated by SDS-PAGE and transferred onto PVDF membranes before being blocked with 5% skim milk at room temperature for 2 h. The membranes were probed overnight at 4°C with primary antibodies. Then, the membrane was re-probed with secondary antibody horseradish peroxidase-labeled goat anti-rabbit IgG (1:2000, ab6721, Abcam) at room temperature for 1 h. The enhanced chemiluminescence reagent (P0018S, Beyotime) was used to visualize the protein bands. The intensities of western blot bands were analyzed with Image J software, normalized to β-actin (1:10000, ab8226, Abcam). The primary antibodies used included SLC31A1 (1:1000, ab317432, Abcam), FDX1 (1:1000, 12592-1-AP, Proteintech, Wuhan, Hubei, China), DLAT (1:1000, 83654-3-RR, Proteintech), NLRP3 (1:1000, 30109-1-AP, Proteintech), cleaved caspase-1 (1:1000, 22915-1-AP, Proteintech), ASC (1:1000, 10500-1-AP, Proteintech), cleaved caspase-3 (1:1000, #9664, CST), Bax (1:1000, 50599-2-Ig, Proteintech), and Bcl-2 (1:1000, 26593-1-AP, Proteintech).

### Biochemical tests

Cu^2+^ and ATP contents, the succinate dehydrogenase (SDH) activity in cells, and the LDH activity in cell supernatants were determined using corresponding kits (Elabscience) (Cu^2+^ assay kit: E-BC-K300-M; ATP assay kit: E-BC-K774-M; SDH assay kit: E-BC-K649-M; LDH assay kit: E-BC-K771-M).

### Enzyme-linked immunosorbent assay

Levels of N-terminal pro-brain natriuretic peptide (NT-proBNP), IL-1β, tumor necrosis factor-α (TNF-α) and HMGB1 were measured in the mouse serum and cell supernatants using ELISA kits (Elabscience) (NT-proBNP kit: E-EL-M0834; IL-1β kit: E-EL-M0037; TNF-α kit: E-EL-M3063; HMGB1 kit: E-EL-M0676).

### Transmission electron microscopy

Following the removal of the cell culture medium, cells were initially rinsed once with PBS and subsequently detached with trypsin (E-EL-SR001, Elabscience). Next, cells were rinsed with a 0.5% glutaraldehyde solution, followed by fixation using 2.5% glutaraldehyde. Subsequent processing involved dehydration, embedding, and sectioning of the samples. Thereafter, sections were stained using standard protocols with lead citrate and uranyl acetate, followed by overnight drying at room temperature. Finally, sections were examined under TEM (JEM-F200, JEOL, Tokyo, Japan) for observation, image acquisition, and subsequent analysis.

### Flow cytometry

Apoptosis of cardiomyocytes was assessed using the Annexin-V-FITC/propidium iodide (PI) double staining apoptosis kit (C1062M, Beyotime). After washing twice with PBS, cells were resuspended in a mixed solution comprising 200 μL binding buffer and stained with Annexin V-FITC/PI for 15 min in the dark. Cell apoptosis was evaluated by a flow cytometer (BD Biosciences) and data were analyzed by FlowJo7.6.1 software (BD Biosciences).

### Statistical analysis

The statistical analysis was performed using GraphPad Prism software (version 9.5.0, GraphPad Software, San Diego, CA, USA). Data were expressed as mean ± standard deviation. One-way analysis of variance (ANOVA) with Tukey’s *post-hoc* test was applied for multi-group data comparisons. *p* < 0.05 in a two-tailed test indicates statistically significant difference.

## Results

### SLC31A1 is preferentially expressed in mice with post-AMI HF, while knockdown of SLC31A1 restrains cardiomyocyte apoptosis and ameliorates HF

**Figure 1:**
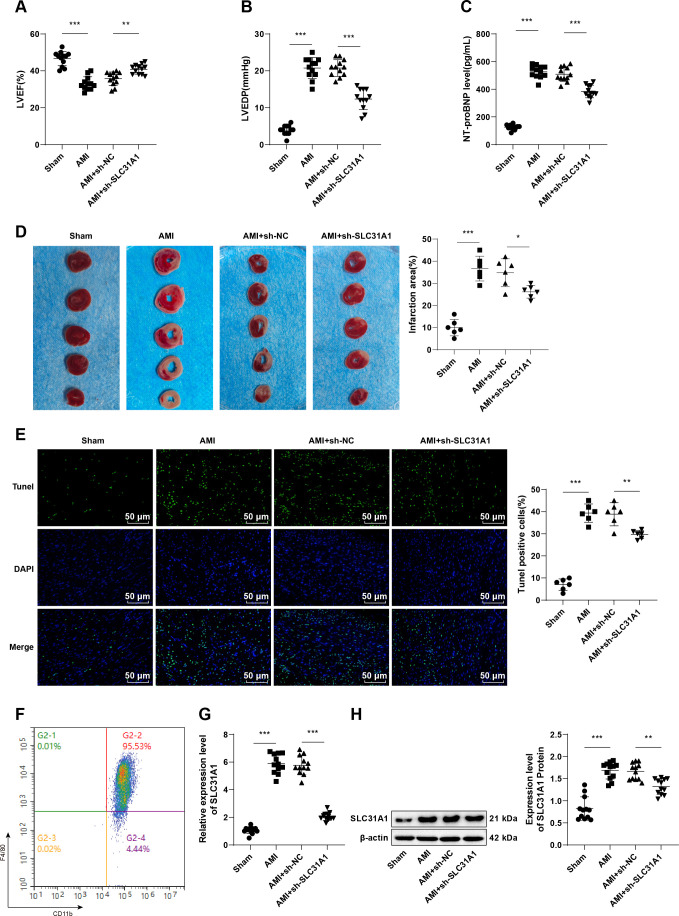
SLC31A1 is abundantly expressed in post-AMI HF mouse models, silencing of which mitigates post-AMI HF by inhibiting cardiomyocyte apoptosis. **(A, B)** LVEF and LVEDP detected by echocardiography, n = 12; **(C)** The serum NT-proBNP level measured by ELISA, n = 12; **(D)** Representative images of TTC-stained heart sections and quantification of myocardial infarction, n = 6; **(E)** Cardiomyocyte apoptosis assessed by TUNEL staining, n = 6; **(F)** Sorting of macrophages (F4/80^+^/CD11b^+^) by flow cytometry; **(G)** mRNA expression of SLC31A1 determined by RT-qPCR, n = 12; **(H)** The level of SLC31A1 protein determined by western blot, n = 3. Data were described as mean ± standard deviation, with multi-group comparisons conducted by one-way ANOVA, followed by Tukey’s *post hoc* tests. *p < 0.05, **p < 0.01, ***p < 0.001.

To explore the specific role of SLC31A1 in post-AMI HF, we first established a post-AMI HF mouse model by ligating the LAD coronary artery. The AMI group showed lower LVEF (*p* < 0.001, **Figure 1A**), higher LVEDP (*p* < 0.001, **Figure 1B**), higher serum NT-proBNP level (*p* < 0.001, **Figure 1C**), larger myocardial infarct size (*p* < 0.001, **Figure 1D**), and higher cardiomyocyte apoptosis (*p* < 0.001, **Figure 1E**) than those of the Sham group. These results demonstrated the successful establishment of the mouse model of post-AMI HF. SLC31A1 has been reported to serve as a biomarker of HF (10), and it is predominantly expressed in macrophages in cardiovascular diseases (13). To this end, in this study, we sorted macrophages (F4/80^+^/CD11b^+^) (**Figure 1F**) from mouse blood by flow cytometry, and then determined the expression of SLC31A1 in the sorted macrophages by RT-qPCR and western blot. Results yielded significantly higher expression of SLC31A1 in macrophages in the AMI group than in the Sham group (all *p* < 0.001, **Figures 1G,H**). Subsequently, adenovirus carrying sh-SLC31A1 was injected into the HF mice. Compared with the AMI + sh-NC group, the AMI + sh-SLC31A1 group had lowered expression of SLC31A1 in mouse blood macrophages (all *p* < 0.001, **Figures 1G, H**), profoundly improved cardiac functions (*p* < 0.01, **Figures 1A–C**), a reduced myocardial infarct size (*p* < 0.05, **Figure 1D**), and decreased cardiomyocyte apoptosis (*p* < 0.01, **Figure 1E**). Collectively, these data suggest that SLC31A1 is abundantly expressed in mouse models of post-AMI HF and its knockdown may alleviate post-AMI HF by suppressing cardiomyocyte apoptosis.

### Knockdown of SLC31A1 regulates copper metabolism imbalance to reduce cuproptosis and HMGB1 release in macrophages, mitigating inflammatory response of mice with post-AMI HF

**Figure 2:**
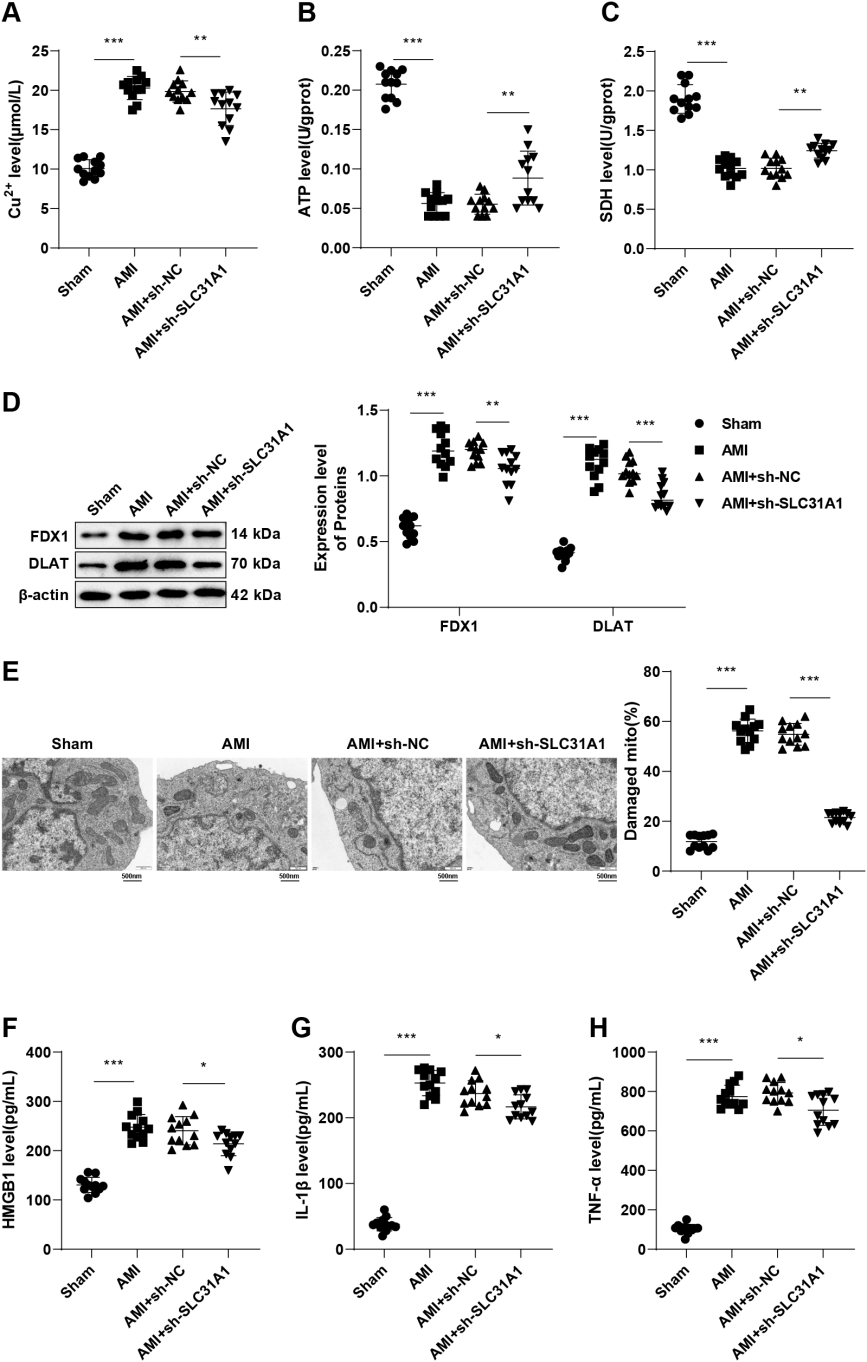
SLC31A1 knockdown arrests cuproptosis and HMGB1 release by regulating copper metabolism imbalance, alleviating inflammatory responses in macrophages of mice with HF after AMI. **(A-C)** Cu^2+^ level, ATP content and SDH activity in mouse macrophages determined by kits; **(D)** Levels of cuproptosis-related proteins FDX1 and DLAT in mouse macrophages measured by western blot; **(E)** Mitochondrial damage in cells detected by TEM; Levels of HMGB1 **(F)**, IL-1β **(G)**, and TNF-α **(H)** in mouse serum measured by ELISA. n = 12. Data were described as mean ± standard deviation, with multi-group comparisons conducted by one-way ANOVA, followed by Tukey’s *post hoc* tests. *p < 0.05, **p < 0.01, ***p < 0.001.

Evidence suggests that SLC31A1 is responsible for cellular Cu^2+^ uptake and is closely linked to cuproptosis (32). The Cu^2+^ content was found to be increased (*p* < 0.001, **Figure 2A**), the ATP content was abated (*p* < 0.001, **Figure 2B**) and the SDH activity was diminished (*p* < 0.001, **Figure 2C**) in mouse macrophages in the AMI group compared with the Sham group, in addition to up-regulated levels of cuproptosis-related proteins FDX1 and DLAT (all *p* < 0.001, **Figure 2D**), severe damaged mitochondria, ruptured outer membrane, and lost cristae (all *p* < 0.001, **Figure 2E**). These results indicate that Cu^2+^ ions accumulated in the macrophages of mice with post-AMI HF, resulting in increased cuproptosis. Upon cuproptosis, HMGB1 can be released into the extracellular space, triggering an inflammatory response (21). Herein, we conducted ELISA to measure levels of HMGB1 and inflammatory factors IL-1β and TNF-α in mouse serum. In the AMI group, their levels were significantly increased relative to the Sham group (all *p* < 0.001, **Figures 2F–H**), suggesting increased release of HMGB1 and resultant inflammatory responses. In addition, it was found that SLC31A1 knockdown led to decreases in Cu^2+^ accumulation and cuproptosis in mouse macrophages (*p* < 0.05, **Figures 2A–E**), as well as diminished serum levels of HMGB1, IL-1β and TNF-α (all *p* < 0.05, **Figures 2F–H**). These experimental results indicate that SLC31A1 knockdown can regulate copper metabolism imbalance, thereby inhibiting cuproptosis and HMGB1 release, eventually effectively improving inflammatory responses in macrophages of mice with post-AMI HF.

### The copper chelator ATTM attenuates macrophage cuproptosis, suppresses cardiomyocyte apoptosis and relieves post-AMI HF in mice

**Figure 3:**
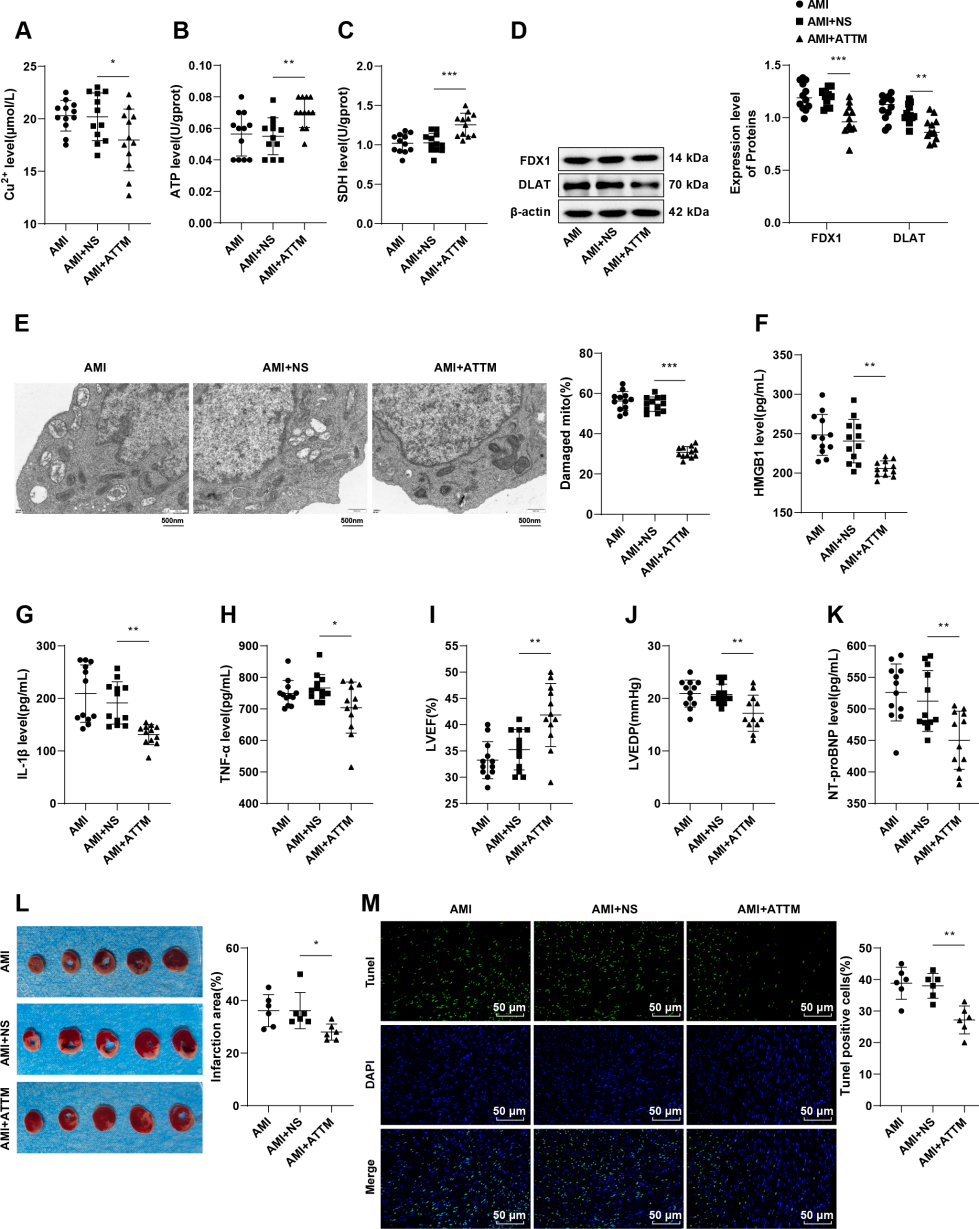
ATTM inhibits cuproptosis in macrophages, impedes cardiomyocyte apoptosis and relieves HF after AMI in mice. **(A-C)** Cu^2+^ level, ATP content, and SDH activity in mouse macrophages determined by kits, n = 12; **(D)** Levels of cuproptosis-related proteins FDX1 and DLAT in mouse macrophages measured by western blot, n = 12; **(E)** Mitochondrial damage in cells assessed by TEM; Levels of HMGB1 **(F)**, IL-1β **(G)**, and TNF-α **(H)** in mouse serum measured by ELISA, n = 12; LVEF **(I)** and LVEDP **(J)** detected by echocardiography, n = 12; K, The NT-proBNP level in mouse serum measured by ELISA, n = 12; L, Representative images of TTC-stained heart sections and quantification of myocardial infarction, n = 6; M, Cardiomyocyte apoptosis assessed by TUNEL staining, n = 6. Data were described as mean ± standard deviation, with multi-group comparisons conducted by one-way ANOVA, followed by Tukey’s *post hoc* tests. *p < 0.05, **p < 0.01, ***p < 0.001.

Based on the aforementioned results, we treated mice with post-AMI HF with the copper chelator ATTM. Compared with the AMI + NS group, the AMI + ATTM group presented a lower Cu^2+^ level (*p* < 0.05, **Figure 3A**), higher ATP content (*p* < 0.01, **Figure 3B**) and SDH activity (*p* < 0.001, **Figure 3C**), as well as down-regulated levels of cuproptosis-related proteins FDX1 and DLAT (all *p* < 0.01, **Figure 3D**), reduced mitochondrial damage (all *p* < 0.01, **Figure 3E**) and serum HMGB1, IL-1β and TNF-α levels (all *p* < 0.05, **Figures 3F–H**). It can be plausible that ATTM could significantly reduce the accumulation of Cu^2+^ ions in mouse macrophages, inhibit cuproptosis and HMGB1 release, and limit inflammatory responses. Furthermore, there was a marked increase in LVEF (*p* < 0.01, **Figure 3I**), and reductions in LVEDP (*p* < 0.01, **Figure 3J**), serum NT-proBNP level (*p* < 0.01, **Figure 3K**), myocardial infarct size (*p* < 0.05, **Figure 3L**), and cardiomyocyte apoptosis (*p* < 0.01, **Figure 3M**) in the AMI + ATTM group relative to the AMI + NS group. Above all, ATTM may have the capacity to improve macrophage cuproptosis, consequently repressing cardiomyocyte apoptosis and ameliorating HF after AMI in mice.

### Knockdown of SLC31A1 or the copper chelator ATTM inhibits cuproptosis and attenuates inflammatory responses in hypoxia-induced macrophages

**Figure 4:**
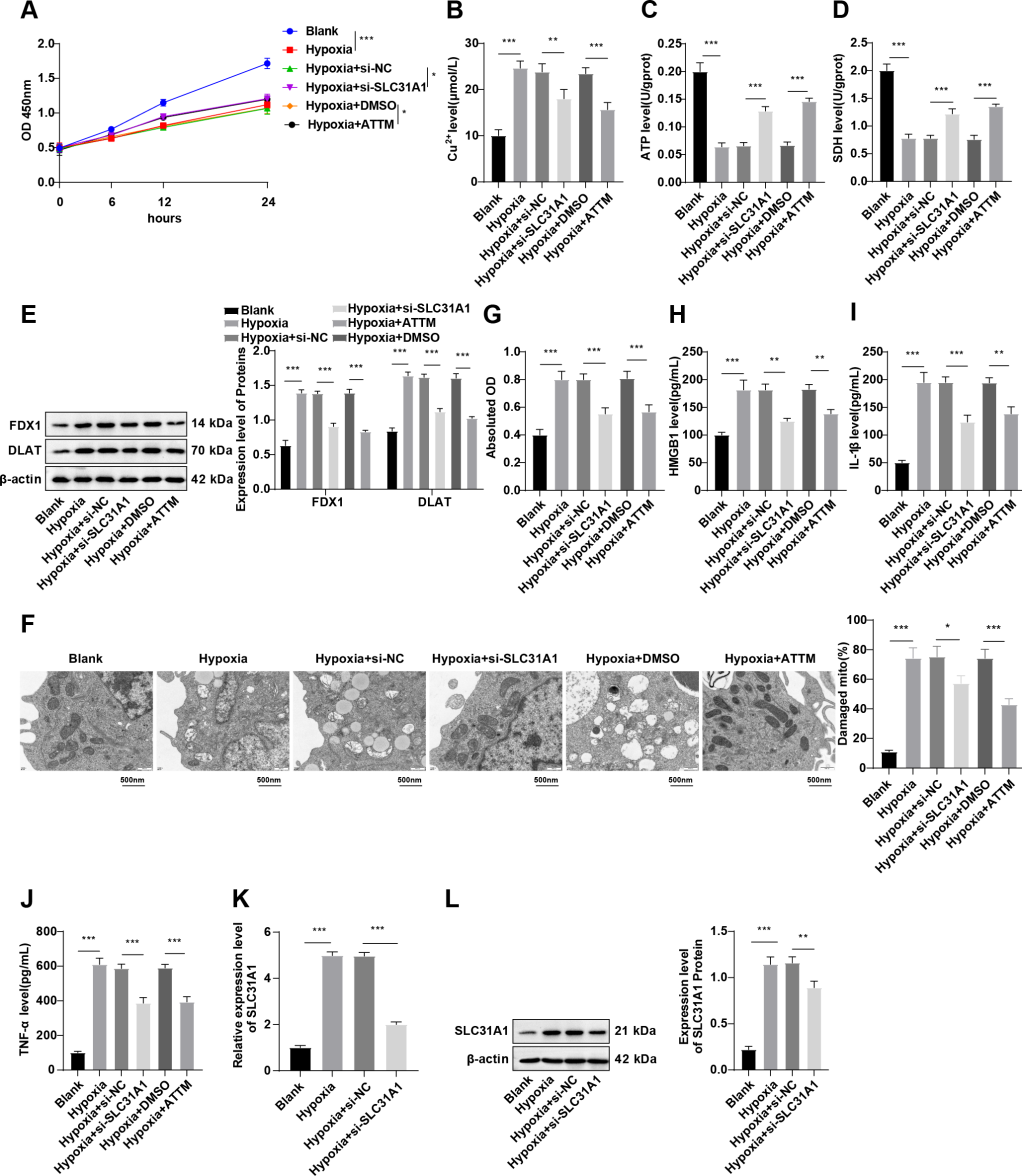
SLC31A1 silencing or ATTM suppresses cuproptosis and inflammatory responses in hypoxia-induced macrophages. **(A)** Cell viability assessed by CCK-8; **(B–D)** The Cu^2+^ level, ATP content, and SDH activity in RAW264.7 cells determined by kits; **(E)** Levels of cuproptosis-related proteins FDX1 and DLAT in RAW264.7 cells measured by western blot; **(F)** Mitochondrial damage in cells assessed by TEM; **(G)** The LDH activity in RAW264.7 cell supernatants detected by the kit; **(H-J)** Levels of HMGB1, IL-1β, and TNF-α in RAW264.7 cell supernatants measured by ELISA; **(K)** SLC31A1 mRNA expression determined by RT-qPCR; **(L)** The SLC31A1 protein level measured by western blot. All cell-based *in vitro* experiments were independently repeated three times. Data were described as mean ± standard deviation, with multi-group comparisons conducted by one-way ANOVA, followed by Tukey’s *post hoc* tests. *p < 0.05, **p < 0.01, ***p < 0.001.

We then centered on characterizing the effect of SLC31A1 or ATTM on macrophage cuproptosis through *in vitro* studies. Specifically, mouse macrophages RAW264.7 were exposed to hypoxia. Relative to the Blank group, the Hypoxia group exhibited lower cell viability (*p* < 0.001, **Figure 4A**), a higher Cu^2+^ level (*p* < 0.001, **Figure 4B**), lower ATP content (*p* < 0.001, **Figure 4C**) and SDH activity (*p* < 0.001, **Figure 4D**), along with higher levels of cuproptosis-related proteins FDX1 and DLAT (all *p* < 0.001, **Figure 4E**), severely damaged mitochondria, ruptured outer membrane and lost cristae (*p* < 0.001, **Figure 4F**), intensified cell death (*p* < 0.001, **Figure 4G**), and increased HMGB1, IL-1β and TNF-α levels (all *p* < 0.001, **Figures 4H–J**). The results indicate that hypoxia induced cuproptosis and release of HMGB1, promoting inflammatory responses in macrophages. Strikingly, treatment with ATTM was observed to partially reverse hypoxia-induced cuproptosis and HMGB1 release, and improve inflammatory responses in macrophages (all *p* < 0.05, **Figures 4A–J**). In addition, after hypoxia induction, expression of SLC31A1 in RAW264.7 cells was significantly increased (all *p* < 0.001, **Figures 4K, L**), while it was diminished upon treatment with the si-SLC31A1 plasmid (all *p* < 0.001, **Figures 4K, L**). Meanwhile, upon SLC31A1 knockdown, cell viability was potentiated (*p* < 0.05, **Figure 4A**), but cuproptosis (all *p* < 0.05, **Figures 4B–G**), and levels of HMGB1, IL-1β, and TNF-α (all *p* < 0.01, **Figures 4H–J**) were repressed. These findings reinforce the notion that SLC31A1 silencing or ATTM may hinder cuproptosis and inflammatory responses in hypoxia-induced macrophages.

### SLC31A1 participates in macrophage cuproptosis and HMGB1 release by regulating activation of the NLRP3 inflammasome

**Figure 5:**
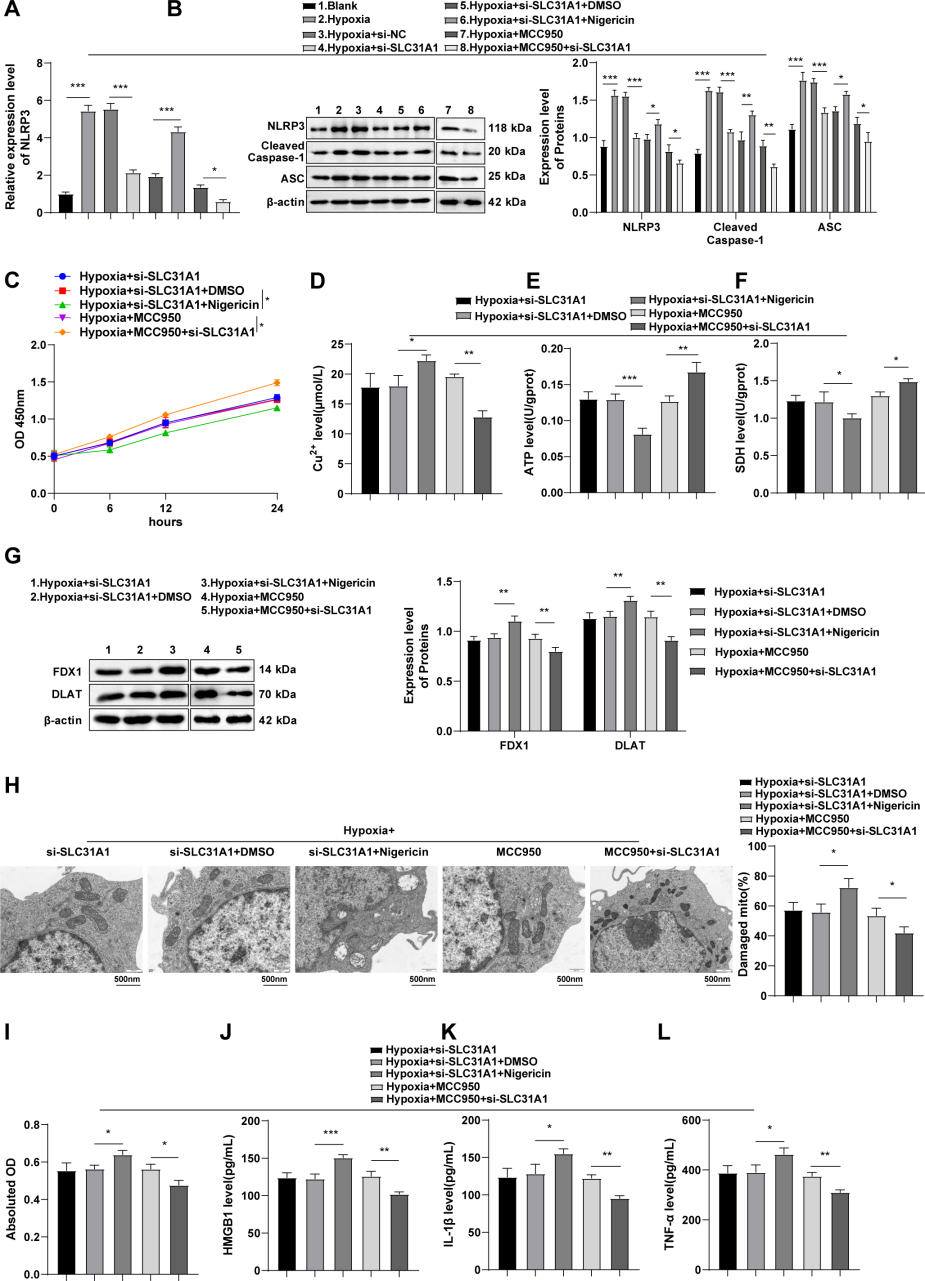
SLC31A1 is implicated in cuproptosis in macrophages through regulation of the NLRP3/HMGB1 pathway. **(A)** NLRP3 mRNA expression determined by RT-qPCR; **(B)** NLRP3, cleaved caspase-1 and ASC protein levels measured by western blot; **(C)** Cell viability evaluated by CCK-8; **(D-F)** The Cu^2+^ level, ATP content, and SDH activity in RAW264.7 cells assessed by kits; **(G)** Levels of cuproptosis-related proteins FDX1 and DLAT in RAW264.7 cells determined by western blot; **(H)** Mitochondrial damage in cells assessed by TEM; **(I)** The LDH activity in RAW264.7 cell supernatants detected by a kit; **(J-L)** Levels of HMGB1, IL-1β, and TNF-α in RAW264.7 cell supernatants measured by ELISA. All cell-based *in vitro* experiments were independently repeated three times. Data were described as mean ± standard deviation, with multi-group comparisons conducted by one-way ANOVA, followed by Tukey’s *post hoc* tests. *p < 0.05, **p < 0.01, ***p < 0.001.

Activation of the NLRP3 inflammasome plays an important role in cuproptosis (33) and the consequent HF (34). In comparison to the Blank group, NLRP3 expression as well as protein levels of cleaved caspase-1 and ASC was evidently up-regulated in the Hypoxia group, but was diminished in the Hypoxia + si-SLC31A1 group versus the Hypoxia + si-NC group (all *p* < 0.001, **Figures 5A, B**). Subsequently, hypoxic RAW264.7 cells were co-treated with si-SLC31A1 and nigericin or MCC950. NLRP3 expression as well as protein levels of cleaved caspase-1 and ASC was partially elevated in the Hypoxia + si-SLC31A1 + Nigericin group when compared with the Hypoxia + si-SLC31A1 + DMSO group (all *p* < 0.01, **Figures 5A, B**). Additionally, co-treatment with si-SLC31A1 and nigericin resulted in arrested cell viability (all *p* < 0.05, **Figure 5C**), enhanced cuproptosis (all *p* < 0.05, **Figures 5D–I**), and increased levels of HMGB1, IL-1β and TNF-α in cell supernatants (all *p* < 0.05, **Figures 5J–L**). Compared to the Hypoxia + MCC950 group, the Hypoxia + MCC950 + si-SLC31A1 group exhibited decreased protein expression of NLRP3, Cleaved Caspase-1, and ASC (all *p* < 0.05, **Figures 5A, B**), increased cell viability (all *p* < 0.05, **Figure 5C**), a subdued cuproptosis level (all *p* < 0.01, **Figures 5D–I**), and diminished HMGB1, IL-1β, and TNF-α levels in the cell supernatant (all *p* < 0.01, **Figures 5J–L**). To conclude, SLC31A1 affects cuproptosis and HMGB1 release in macrophages by activating the NLRP3 inflammasome. Moreover, knockdown of SLC31A1 can disrupt the NLRP3/HMGB1 pathway and contribute to cuproptosis inhibition.

### Knockdown of SLC31A1 perturbs cardiomyocyte apoptosis through the NLRP3/HMGB1-dependent macrophage cuproptosis pathway

**Figure 6:**
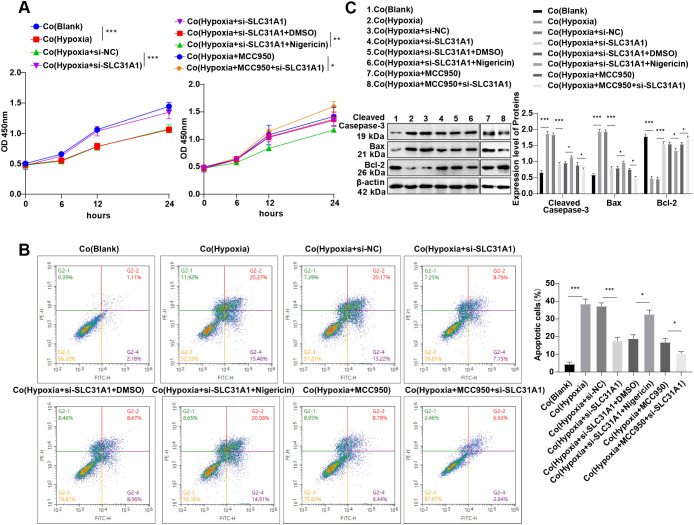
SLC31A1 silencing hampers cardiomyocyte apoptosis via the NLRP3/HMGB1-dependent macrophage cuproptosis pathway. **(A)** H9C2 cell viability assessed by CCK-8; **(B)** H9C2 cell apoptosis analyzed by flow cytometry; **(C)** Cleaved caspase-3, Bcl-2 and Bax protein levels in H9C2 cells determined by western blot. All cell-based *in vitro* experiments were independently repeated three times. Data were described as mean ± standard deviation, with multi-group comparisons conducted by one-way ANOVA, followed by Tukey’s *post hoc* tests. *p < 0.05, **p < 0.01, ***p < 0.001.

HMGB1 is an inflammatory mediator to provoke cardiomyocyte apoptosis (22). In the current study, we co-cultured cardiomyocyteswithRAW264.7 cells from each group. We found decreased cardiomyocyte viability (all *p* < 0.001, **Figure 6A**), and remarkably enhanced cell apoptosis (all *p* < 0.001, **Figure 6B**), accompanied by elevated levels of apoptosis-related proteins cleaved caspase-3 and Bax and a declined Bcl-2 level (all *p* < 0.001, **Figure 6C**) in the Co(Hypoxia) group, when compared with the Co(Blank) group. Interestingly, cardiomyocyte viability was higher in the Co(Hypoxia + si-SLC31A1) group than in the Co(Hypoxia + si-NC) group (*p* < 0.001, **Figure 6A**), while cell apoptosis (all *p* < 0.001, **Figure 6B**), and the levels of related proteins cleaved caspase-3 and Bax were diminished (all *p* < 0.001, **Figure 6C**), in addition to an increased Bcl-2 level (all *p* < 0.001, **Figure 6C**). In these cases, we can conclude that SLC31A1 knockdown in hypoxia-induced macrophages may fuel cardiomyocyte viability and impede apoptosis. Moreover, co-treatment with si-SLC31A1 and nigericin caused a remarkable decrease in cardiomyocyte viability and apoptosis induction (all *p* < 0.05, **Figures 6A–C**). Knockdown of SLC31A1 and simultaneous suppression of the NLRP3 pathway resulted in an elevation in cardiomyocyte viability and a reduction in apoptosis (all *p* < 0.05, **Figures 6A–C**). Collectively, our findings validate that SLC31A1 knockdown can impair cardiomyocyte apoptosis by blocking the NLRP3/HMGB1-dependent macrophage cuproptosis pathway.

### Activation of the NLRP3/HMGB1 pathway partly negates SLC31A1 knockdown-induced alleviation of macrophage cuproptosis and cardiomyocyte apoptosis in mice with post-AMI HF

**Figure 7:**
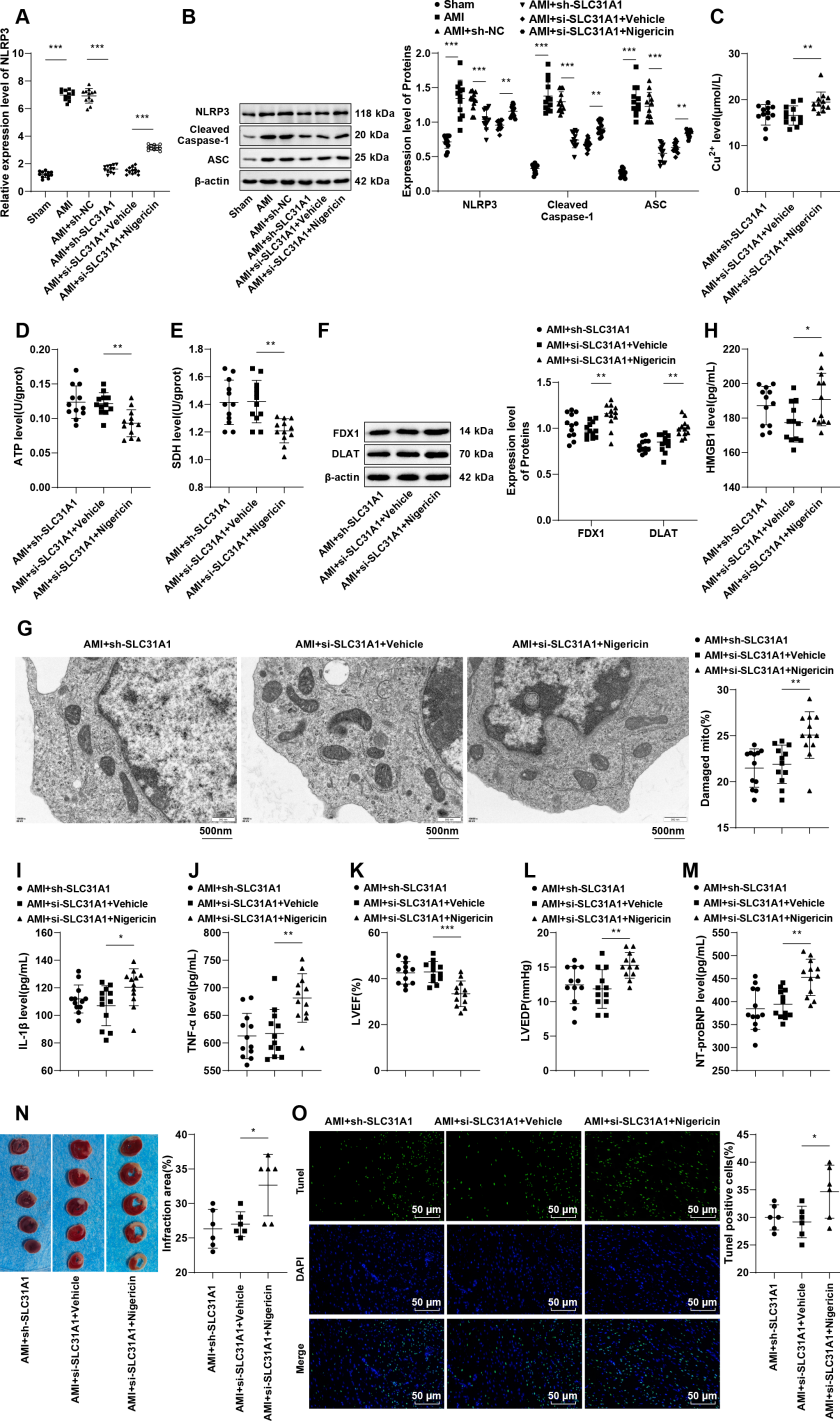
Activation of the NLRP3/HMGB1 pathway partly counteracts the protection against macrophage cuproptosis and cardiomyocyte apoptosis mediated by SLC31A1 knockdown in mice with post-AMI HF. **(A)** NLRP3 mRNA expression in mouse macrophages determined by RT-qPCR, n = 12; **(B)** NLRP3, cleaved caspase-1 and ASC protein levels in mouse macrophages measured by western blot, n = 12; **(C-E)** The Cu^2+^ level, ATP content, and SDH activity in mouse macrophages assessed by kits, n = 12; **(F)** Levels of cuproptosis-related proteins FDX1 and DLAT in mouse macrophages determined by western blot, n = 12; **(G)** Mitochondrial damage in cells assessed by TEM; **(H-J)** Levels of HMGB1, IL-1β, and TNF-α in mouse serum measured by ELISA, n = 12; **(K-L)** LVEF and LVEDP detected by echocardiography, n = 12; **(M)** The NT-proBNP level in mouse serum measured by ELISA, n = 12; **(N)** Representative images of TTC-stained heart sections and quantification of myocardial infarction, n = 6; **(O)** Cardiomyocyte apoptosis evaluated by TUNEL staining, n = 6. Data were described as mean ± standard deviation, with multi-group comparisons conducted by one-way ANOVA, followed by Tukey’s *post hoc* tests. *p < 0.05, **p < 0.01, ***p < 0.001.

Finally, we conducted *in vivo* experiments in mice to validate the function of NLRP3. The HF mice were injected with adenovirus carrying sh-SLC31A1 and treated with nigericin simultaneously. It was noted that NLRP3 mRNA and protein levels, along with cleaved caspase-1 and ASC protein levels, were higher in macrophages of mice in the AMI group than in the Sham group, yet opposite results were noticed in the AMI + sh-SLC31A1 group relative to the AMI + sh-NC group (all *p* < 0.001, **Figures 7A, B**). In the AMI + sh-SLC31A1 + Nigericin group, we observed up-regulated levels of NLRP3 mRNA and protein, cleaved caspase-1 and ASC proteins (all *p* < 0.01, **Figures 7A, B**), cuproptosis (all *p* < 0.05, **Figures 7C–G**), and serum HMGB1, IL-1β, and TNF-α (all *p* < 0.05, **Figures 7H–J**), an aggravated cardiac dysfunction (*p* < 0.05, **Figures 7K–M**), an increased myocardial infarct size (*p* < 0.05, **Figure 7N**), and enhanced cardiomyocyte apoptosis (*p* < 0.05, **Figure 7O**) versus the AMI + sh-SLC31A1 + Vehicle group. Altogether, NLRP3 activation can partly undermine the ameliorating effects of SLC31A1 knockdown on macrophage cuproptosis and cardiomyocyte apoptosis in mice with post-AMI HF.

## Discussion

AMI, a major global cardiovascular disease, affects millions annually and recurs in over 50% of patients (35, 36). Despite medical advances, AMI morbidity and mortality, particularly among the young, have shown limited improvement in recent decades (37). As a common complication of AMI, HF adversely affects both short- and long-term patient survival (38). Notably, the novel cell death pathway, cuprotosis, has been implicated in HF pathogenesis, with the cuprotosis marker SLC31A1 playing a significant role (12). This study first reveals that SLC31A1 may mediate copper metabolism imbalance and provoke cuproptosis in macrophages by activating the NLRP3 inflammasome, which enables HMGB1 release, and the subsequent inflammatory response and cardiomyocyte apoptosis. Eventually, post-AMI HF is accelerated. These promising results provide some insights regarding the utility of targeting SLC31A1 as a novel strategy in the management of post-AMI HF and the associated cardiovascular diseases.

Copper metabolism denotes a complex dynamic process regulated by various molecules; the uptake of copper ions is mainly mediated by the copper transporter SLC31A1 in addition to SLC31A2. When extracellular copper levels rise, SLC31A1 is removed from the cell membrane via internalization to reduce excessive copper uptake and prevent copper toxicity. Conversely, when copper levels decrease, SLC31A1 is transported back to the cell surface via a retromer-mediated recycling pathway to enhance copper uptake and maintain intracellular copper balance (39). A major finding of the current study was the increased SLC31A1 expression in mice with post-AMI HF. This was supported by recent findings that SLC31A1 is aberrantly overexpressed in the context of AMI (10). Mitochondrial function and integrity are essential for cardiomyocyte survival; in cardiovascular diseases, a moderate copper level can maintain mitochondrial function to sustain normal cardiac and blood vessel activity, but cuproptosis due to copper overload is positioned to be a critical factor underlying alterations in mitochondrial enzymes (40–42). Abnormal SLC31A1 expression leads to decreased antioxidant enzyme activity, raised oxidative stress, and an impaired mitochondrial function, thereby facilitating the onset and progression of cardiomyopathy (43). Furthermore, downregulation of SLC31A1 depletes mitochondrial copper, hinders oxidative phosphorylation, and promotes cardiac fibrosis (44). In MI, cardiomyocyte apoptosis following impaired mitochondrial homeostasis underlies the fundamental cause of cardiomyocyte loss, progressive cardiac dysfunction, ventricular remodeling and subsequent progression of HF (45, 46). Innovatively, our study results revealed a profound decrease of cardiomyocyte apoptosis in post-AMI HF mice following SLC31A1 knockdown. Much in line with our results, previous findings have demonstrated that upregulation of SLC31A1 disturbs copper homeostasis and promotes cuproptosis, further leading to exacerbated apoptosis in cardiomyocytes in myocardial injury (47, 48). Hence, knockdown of SLC31A1 may represent a viable target for therapeutic intervention to weaken cardiomyocyte apoptosis and subdue the progression of post-AMI HF.

Moreover, in our study, the copper chelator ATTM was observed to have the potency to obstruct macrophage cuproptosis, decelerate cardiomyocyte apoptosis and improve HF after AMI in mice. This is consistent with previous evidence suggesting that ATTM confers cardio-protection in a porcine model of AMI and reperfusion in a drug-exposure-dependent manner, which is evidenced by a lowered blood troponin T level and an elevated activity of myocardial glutathione peroxidase, an antioxidant selenoprotein, in ATTM-treated animals (49). Besides, cuproptosis inducer elesclomol plus copper chloride significantly reduces ATP production, weakens the activity of mitochondrial complex I and III, inhibits cardiomyocyte viability, and increases contents of injury markers LDH, MDA, and cTnI, eventually resulting in cardiomyocyte injury; however, these effects are abolished by the copper chelator ATTM (50, 51), The copper chelator triethylenetetramine also has been documented to be used in patients with diabetic cardiomyopathy and HF. Reportedly, it can improve left ventricular structure and function and restore copper metabolism balance. Moreover, a Phase II clinical trial for HF is underway (52). If the mechanism of copper chelators can be further clarified, dosing regimens optimized, and validated through clinical trials, copper chelators may become an adjunctive treatment for MI.

HMGB1 is a key immune mediator of cuproptosis-initiated inflammation; specifically, copper accumulation promotes HMGB1 phosphorylation and the consequent extracellular release *via* ATP elimination and AMPK activation; upon release, HMGB1 functions as a damage-associated molecular pattern protein to trigger immune responses by binding multiple pattern recognition receptors, such as advanced glycosylation end product-specific receptor and toll-like receptor 4 (21, 53). Based on our experimental results, knockdown of SLC31A1 can modulate copper metabolism imbalance, reduce cuproptosis and HMGB1 release in macrophages of HF mice, thereby effectively ameliorating the inflammatory response. Indeed, the NLRP3 inflammasome activation can facilitate the release of HMGB1 by macrophages (19, 54, 55) and promote the secretion of inflammatory factors, thus exacerbating cardiac inflammation (56). Moreover, NLRP3 is closely related to cuproptosis and has been identified as a cuproptosis-related biomarker (57, 58). Our study for the first time discovered that SLC31A1 could participate in regulating macrophage cuproptosis and HMGB1 release by activating the NLRP3 inflammasome and that knockdown of SLC31A1 impeded the NLRP3/HMGB1 pathway and improved macrophage cuproptosis. Besides cuproptosis, NLRP3-mediated ferroptosis also plays a role in myocardial injury (59). Cuproptosis is characterized by mitochondrial proteotoxicity, while ferroptosis is characterized by lipid peroxidation (60). Both processes in HF are driven by disordered metal metabolism and oxidative stress, and exhibit cross-talk and synergistic effects. It has been reported that copper overload can not only induce cuproptosis but also may induce ferroptosis through mechanisms such as enhancement of ROS generation and inhibition of GPX4, forming a positive feedback injury loop (8). Besides participating in copper metabolism regulation via the NLRP3/HMGB1 pathway, SLC31A1 can also influence copper metabolism dysregulation and copper precipitation via the KDM5B/FTH1 axis (61). Furthermore, various pathways, including copper transport (39), epigenetic modifications (44), and the p62-EZH2/β-catenin-TCF4 axis (62, 63), may regulate the SLC31A1 level to influence myocardial injury by affecting mitochondrial function (43), glycolysis (44), pyroptosis (64), and autophagy (65). Taken together, results of our studies and previous reports provide strong evidence to support that SLC31A1 knockdown perturbed cardiomyocyte apoptosis through inhibiting the NLRP3/HMGB1-dependent macrophage cuproptosis.

In summary, our study for the first time revealed the SLC31A1/NLRP3/HMGB1 signaling axis may represent a novel therapeutic target for the future clinical management of post-AMI HF and other lethal cardiovascular diseases. However, there are several limitations warrant further investigation. First, although this study provides mechanistic evidence, the long-term therapeutic effects of SLC31A1 knockdown across various animal models still require further validation. Given that SLC31A1 knockdown improves HF, future efforts could focus on developing SLC31A1-targeted small molecule drugs or AAV-based therapeutic strategies and on validating the findings in large animal models or pre-clinical trials. Second, although this study found that copper deficiency leads to cuproptosis and HMGB1 release in macrophages, thereby promoting cardiomyocyte apoptosis, the specific metabolic pathways, the extent of impact on oxidative phosphorylation, and the dynamic process of energy metabolism changes warrant further exploration. Third, although ATTM and gene knockdown technology have achieved great success in experiments, their clinical applications face many challenges, including specificity, safety and effectiveness of intervention drugs.

## Data Availability

All data generated or analysed during this study are included in this article. Further enquiries can be directed to the corresponding author.
